# Tumor markers and depression scores are predictive of non-suicidal self-injury behaviors among adolescents with depressive disorder: A retrospective study

**DOI:** 10.3389/fnins.2022.953842

**Published:** 2022-08-11

**Authors:** Peng-cheng Yi, Yan-hua Qin, Chun-mei Zheng, Ke-ming Ren, Lei Huang, Wei Chen

**Affiliations:** ^1^Department of Psychiatry, Sir Run Run Shaw Hospital, Zhejiang University School of Medicine, Hangzhou, China; ^2^Department of Clinical Psychology, The Third People’s Hospital of Xiangshan County, Ningbo, China; ^3^Department of Psychiatry, The Seventh People’s Hospital of Shaoxing, Shaoxing, China; ^4^Department of Psychology and Behavioral Sciences, Zhejiang University, Hangzhou, China; ^5^Key Laboratory of Medical Neurobiology of Zhejiang Province, Hangzhou, China

**Keywords:** depression disorder, adolescent, non-suicidal self-injury behavior, tumor marker, predictive index

## Abstract

**Background:**

Non-suicidal self-injury (NSSI) is an important risk factor for suicide in adolescents with depressive disorders; therefore, it is important to predict NSSI occurrence as early as possible. Disturbances in biological rhythms are characteristic manifestations of depressive disorders and can lead to immune dysfunction, leading to changes in tumor markers. This study aimed to produce an index that utilizes tumor markers to predict NSSI behaviors among adolescents with depressive disorders.

**Methods:**

A total of 120 hospitalized adolescent patients with depressive disorders aged 14–24 years were included in this study. Participants were divided into NSSI and non-NSSI groups based on self-reports using the Ottawa Self-Injury Inventory. Demographics, tumor marker concentrations, other peripheral blood indices, Hamilton Depression Rating Scale (HDRS) scores, and Hamilton Anxiety Rating Scale (HAMA) scores were compared between the two groups. Logistic regression analysis was conducted to develop a joint index, and a receiver operating characteristic (ROC) curve was created to predict NSSI behaviors among adolescents with depressive disorders.

**Results:**

Compared with the non-NSSI group, the NSSI group had significantly higher insight, retardation, insomnia, hopelessness, psychiatric anxiety, total HDRS and HAMA scores, and significantly higher levels of cancer antigen 125 (CA-125), cancer antigen 19-9 (CA19-9), and carcinoembryonic antigen (CEA). In addition, a joint index was developed by combining CA-125, CA19-9, CEA, HDRS total score, HAMA total score and age using multiple logistic regression to predict NSSI behaviors. The area under the curve was 0.831, with a sensitivity and specificity of 0.734 and 0.891, respectively.

**Conclusion:**

A combination of depression score, tumor marker levels, and age can identify NSSI behaviors among adolescents with depressive disorders.

## Introduction

The prevalence of depressive disorders in adolescents has been gradually increasing, making it the major disorder leading to adolescent disability and a major cause of adolescent suicide (Gore et al., 2011; Zubrick et al., 2017; Tang et al., 2019; Daly, 2022). The lifetime suicide rate for untreated depressive disorder is approximately 20%, and the total suicide mortality rate has reached 3.33 per 100,000 in adolescents aged 10–24 years (Sawyer et al., 2018; Dome et al., 2019). To prevent adolescents from performing suicidal behavior (SB), suicide risk factors need to be studied for targeted prevention and intervention.

Non-suicidal self-injury (NSSI) refers to a series of behaviors that will not lead to death, such as cutting, skin burning, and hitting, that deliberately and directly harm an individual’s own body without suicidal intent (Lloyd-Richardson et al., 2007). NSSI is considered an independent risk factor for suicidal thoughts and behaviors, and approximately 24% of adolescents report performing NSSI annually (Giletta et al., 2012; Guan et al., 2012). Among all NSSI cases, 70% report per forming NSSI in the past year, and 6.7% meet the diagnostic criteria for potential NSSI disorders according to the Diagnostic and Statistical Manual of Mental Disorders (5th ed.; DSM-5) (American Psychiatric Association, 2013; Zetterqvist et al., 2013; Brausch and Muehlenkamp, 2018). The risk of suicide in adolescents with depressive disorders increases 1.7-fold if they have NSSI behaviors (Groschwitz et al., 2015). Furthermore, as NSSI frequency increases, the proportion of adolescents with suicidal ideation (SI) and attempted suicides (AS) increases 2–4-fold within a year (Zubrick et al., 2017). A previous meta-analysis indicates that the frequency of NSSI and the number of NSSI methods are the strongest predictors of AS after the occurrence of SI (Victor and Klonsky, 2014). Other studies indicate that the incidence rate of NSSI among young people without self-reported major depressive disorder (MDD) (4.4%) is much lower than that in adolescents with self-reported MDD (46.6%) (Zubrick et al., 2016). The same conclusion was drawn regarding depressive disorders that were vulnerable to NSSI (Fox et al., 2015). Therefore, it is important to investigate predictors of NSSI in adolescents with depressive disorders for clinical risk assessment and therapeutic interventions (Fan et al., 2021).

A study of 91,000 individuals indicates a potential association between biological rhythm disorders and MDD or bipolar disorder (BD) (Lyall et al., 2018). In fact, sleep disorder, a type of biological rhythm disorder, is a key feature of depressive disorders. Similar to individuals with biological rhythm disorders, many individuals with depression have sleep rhythm disturbances, poor mood in the morning, improved mood in the evening, and delayed peaks of melatonin and glucocorticoid levels compared to healthy individuals (Horstmann and Binder, 2011; Tan and Li, 2016; Crouse et al., 2021). Studies have demonstrated that biological rhythm disorders can lead to immune dysfunction and destroy macrophage rhythms such that the number of proinflammatory cytokines and tumor necrosis factors in the frontal lobe of suicidal adolescents increases significantly (Pandey et al., 2012). The level of proinflammatory cytokines and tumor necrosis factors in the peripheral blood of NSSI patients observed in the laboratory is higher and is positively correlated with frontal electroencephalography activity, suggesting that inflammation affects frontal lobe dysfunction, increases impulsive traits, and leads to NSSI recurrence (Kim et al., 2020). In addition, some inflammatory factor changes, such as decreased interleukin-8 and increased white blood cell count (WBC), are also associated with suicide and NSSI (Keaton et al., 2019; Iznak et al., 2021). This change in immune function promotes the occurrence and development of chronic inflammatory diseases including cancer and diabetes (Timmons et al., 2021). Furthermore, specific circadian rhythm disorders are a common feature of cancer (Sancar and VanGelder, 2021).

Given that patients with depressive disorders have disturbed biological rhythms that lead to impaired immune function and promote the development of chronic inflammatory diseases, we hypothesized that a significant difference in tumor marker levels would be found between the NSSI and non-NSSI groups of adolescents with depressive disorders. Combined with clinical characteristics, NSSI predictors can be established. Therefore, we retrospectively collected and analyzed tumor markers, psychiatric status, and clinical indicators of adolescents with MDD and BD. This study aimed to develop an index using tumor markers and depressive scores to predict NSSI behaviors among adolescents with depressive disorders.

## Materials and methods

### Study participants

The Institutional Review Board of Sir Run Run Shaw Hospital, School of Medicine, Zhejiang University, approved this retrospective study (No. 20191203-13). The study adhered to the principles of the Declaration of Helsinki. Informed consent was obtained from all participants involved in the study.

A total of 120 hospitalized adolescent patients with depressive disorders in the Department of Psychiatry from July 2016 to July 2019 were included in this study. The inclusion criteria were as follows: (1) confirmed diagnosis of a depressive disorder (i.e., MDD or BD) according to the DSM-5 (American Psychiatric Association, 2013); (2) total score on the 17-item Hamilton Depression Scale (HDRS-17) ≥ 10 points (Hamilton, 1960); and (3) between 14 and 24 years of age (Zubrick et al., 2017). Patients were excluded if (1) they were diagnosed with additional mental disorders; (2) they were diagnosed with a severe physical disease or medical condition including hyperthyroidism, liver and kidney cyst, ovarian cyst, breast hyperplasia, autoimmune disease, infection, severe systemic disease, epilepsy, diabetes, hypertension or other cardiovascular diseases, or obvious liver and kidney disease; (3) they had an intellectual disability or neurodevelopmental disorder, traumatic brain injury, or history of major surgery; (4) they had a history of alcohol, tobacco, or other substance dependence or abuse; or (5) they were pregnant or currently menstruating.

### Clinical assessment

HDRS-17 assesses the depressive symptoms of patients, including (1) anxiety and somatization, (2) weight loss, (3) cognitive disorder, (4) diurnal change, (5) block, (6) sleep disorder, and (7) sense of despair. Most items of HDRS adopt the five-level scoring method of 0−4 points (0: none; 1: mild; 2: moderate; 3: severe; 4: very heavy), and a few items adopt the three-level scoring method of 0−2 points (0: none; 1: suspicious or slight; 2: obvious symptoms). The higher the score, the worse the depressive symptoms. The reliability and validity of HDRS were 0.88−0.99 and 0.92, respectively (Hamilton, 1960).

Hamilton Anxiety Rating Scale (HAMA) was used to assess patients’ anxiety symptoms, including somatic and mental anxiety. All items of HAMA were scored by five grades of 0−4. The criteria of each grade were: (0) asymptomatic; (1) light; (2) medium; (3) heavy; and (4) extremely heavy. The higher the score, the worse the anxiety symptoms. The reliability and validity of HAMA were 0.92 and 0.979, respectively (Hamilton, 1969).

The Ottawa self-injury Inventory (OSI) in the Chinese version is a self-assessment scale, which consists of 28 items. It is used to assess the frequency of NSSI and suicide in the last 1, 6, and 12 months and prior to 1 year ago, the age of onset, the source and concealment of self-injury thoughts, the feeling of self-injury impulse, the location, mode and motivation of the first and current self-injury, the role of self-injury in releasing negative emotions, and the time interval between self-injury thoughts and implementation actions. The correlation between self-injury and stress events, potential addictive characteristics, resistance strategies, and seeking treatment were assessed by Likert two (yes and no) and five (0, 1, 2, 3, and 4) grades. The reliability and validity of the OSI Chinese version were 0.952 and 0.755, respectively (Zhang et al., 2015).

### Peripheral blood index collection

Detailed clinical characteristics and laboratory test data were collected from electronic medical records. Results of routine blood, liver and renal function, blood lipid level, electrolyte level, thyroid function, and sex hormone level tests were collected on the day of admission. These indicators included in this study were WBC, absolute neutrophil count (ANC), percentage of ANC in WBC, absolute lymphocyte count (ALC), hemoglobin (Hb), red blood cell count (RBC), platelet count (PLT), serum albumin (ALB), C-reactive protein (CRP), alanine aminotransferase (ALT), aspartate aminotransferase (AST), creatinine, urea nitrogen, thyroxine, thyrotropin, alpha fetoprotein (AFP), cancer antigen 125 (CA-125), cancer antigen 19-9 (CA19-9), and carcinoembryonic antigen (CEA). Samples for these markers were collected in the morning and examined by laboratory physicians who were blinded to the clinical status of the participants.

### Statistical analysis

Continuous variables were expressed as mean ± standard deviation or median and interquartile range for normally and non-normally distributed variables, respectively. Categorical variables were described as frequencies and percentages. Numerical differences between the two groups were assessed using the chi-square test for categorical variables and the *t*-test or Mann–Whitney *U* test for continuous variables. Least significant difference was used for pairwise comparison between groups in multiple comparisons.

Spearman correlation analysis was performed between NSSI frequency and tumor markers at different time points to assess their relationship. The receiver operating characteristic (ROC) curve and area under the curve (AUC) were used to evaluate the ability of tumor markers to predict the occurrence of NSSI. The Youden index was developed for the optimal cutoff value, and the sensitivity and specificity were calculated. Multiple stepwise logistic regression was conducted to screen for significant factors associated with NSSI, and a joint index was constructed. The ROC curve and AUC of this joint index were further calculated to determine its ability to predict the occurrence of NSSI, and the bootstrap validation model was used to calculate the 95% interval of AUC with 100 self-samplings for validation.

The threshold for significance was set at *P* = 0.05. All statistical analyses were conducted using SPSS, Version 22.0 (IBM Corporation, Armonk, NY, United states), and R software, Version 3.6 (Microsoft Corporation, Seattle, WA, United states).

## Results

### Demographics and clinical characteristics

**FIGURE 1:**
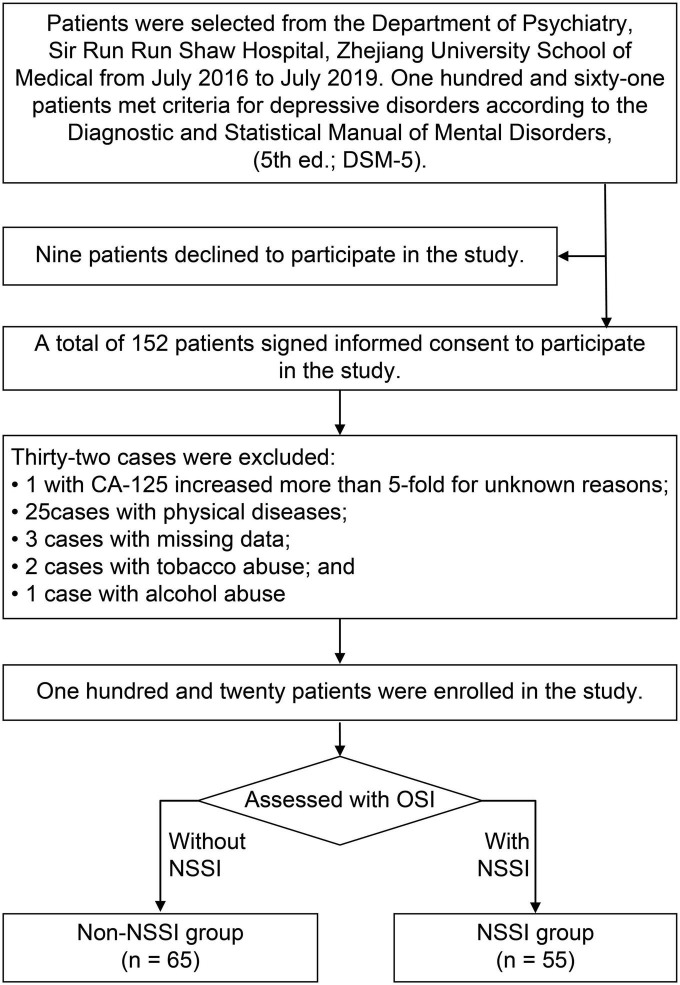
Study flowchart.

First, we screened 161 patients diagnosed with depressive disorders. As shown in Figure 1, nine were excluded because they refused to sign consent forms. We also excluded 32 cases, including one case in which CA-125 increased more than five-fold for unknown reasons, three cases that had missing data, 25 cases with physical diseases (five with breast nodules, two with ovarian cysts, four with liver cysts, one with renal cysts, five with thyroid dysfunction, one with an abnormal brain CT scan result, two with type 1 diabetes, two with severe malnutrition, one with intellectual disability, and two with epilepsy), two cases of tobacco abuse, and one case of alcohol abuse.

Finally, a total of 120 patients were enrolled in the analysis, including 55 in the NSSI group and 65 in the non-NSSI group. The demographic and clinical characteristics of the two groups are shown in Table 1. No significant differences were found in sex, age, disease duration, education level, diagnosis, marital status, residence, or occupation (*p* > 0.05).

**TABLE 1:** Demographic information.

Item	NSSI group (*n* = 55)	Non-NSSI group (*n* = 65)	Z/χ^2^	*P*-value
Sex (M/F, n)	20/35	34/31	3.060	0.098
Age (years)	18.81 ± 3.06	19.71 ± 2.92	−1.525	0.127
Disease duration (months)	14.89 ± 8.19	16.43 + 8.02	−1.158	0.247
Years of education	11.47 ± 3.47	12.27 ± 3.48	−1.185	0.236
Diagnosis			3.238	0.198
BD-type I (n)	4	11		
BD-type II (n)	22	19		
MDD (n)	29	35		
Marital status			0.194	0.999
Unmarried (n)	54	63		
Married (n)	1	2		
Residence			0.656	0.435
Urban (n)	19	18		
Rural (n)	36	47		
Occupation			3.055	0.095
Student (n)	49	50		
Other (n)	6	15		

Sex, marital status, residence, and occupation are displayed as absolute counts; age, disease duration, and years of education were recorded on the day of admission. ANOVAs were performed for sex, diagnosis, marital status, and residence. Mann–Whitney rank-sum tests for two independent samples were performed on age, disease duration, and years of education. BD, bipolar disorder; F, female; M, male; MDD, major depressive disorder; NSSI, non-suicidal self-injury.

### Comparison of hamilton depression rating scale and hamilton anxiety rating scale

Compared with the non-NSSI group, the NSSI group had significantly higher total HDRS scores (*Z* = 5.465, *p* < 0.001) and higher factor scores regarding insight (*Z* = 4.547, *p* < 0.001), diurnal variation (*Z* = 2.179, *p* = 0.029), retardation (*Z* = 3.477, *p* < 0.001), insomnia (*Z* = 2.056, *p* = 0.040), and hopelessness (*Z* = 3.228, *p* = 0.001). In addition, the total HAMA (*Z* = 2.863, *p* = 0.004) and psychiatric symptoms of anxiety scores (*Z* = 2.931, *p* = 0.002) were also significantly higher in the NSSI group than those in the non-NSSI group (Table 2). These results suggest that depression and anxiety symptoms were more severe in the NSSI group.

**TABLE 2:** HDRS and HAMA scores.

Item	NSSI group (*n* = 55)	Non-NSSI group (*n* = 65)	*Z*	*P*-value
HDRS score	22.56 ± 7.56	16.35 ± 4.76	5.465	<0.001
Anxiety, somatic symptoms	3.75 ± 3.48	2.85 ± 1.68	1.458	0.145
Weight loss	0.62 ± 0.93	0.75 ± 0.98	0.854	0.393
Insight	4.44 ± 2.47	2.48 ± 1.98	4.547	<0.001
Diurnal variation	1.64 ± 1.28	1.14 ± 1.10	2.179	0.029
Retardation	5.13 ± 2.29	3.65 ± 1.84	3.477	0.001
Insomnia	2.55 ± 1.58	1.68 ± 1.32	2.056	0.040
Hopelessness	3.80 ± 1.92	2.68 ± 1.59	3.228	0.001
Somatic symptoms, general	0.95 ± 0.78	1.14 ± 1.04	0.793	0.428
HAMA score	12.73 ± 8.09	8.80 ± 7.02	2.863	0.004
Anxiety, somatic symptoms	3.76 ± 4.23	2.62 ± 3.14	1.480	0.139
Anxiety, psychiatric symptoms	8.72 ± 4.76	6.18 ± 4.41	3.147	0.002

The Hamilton Depression Scale (HDRS) and Hamilton Anxiety Scale (HAMA) were assessed on the day of admission. Mann–Whitney rank-sum tests for two independent samples were conducted for all statistical analyses. NSSI, non-suicidal self-injury.

### Comparison of peripheral blood index

**FIGURE 2:**
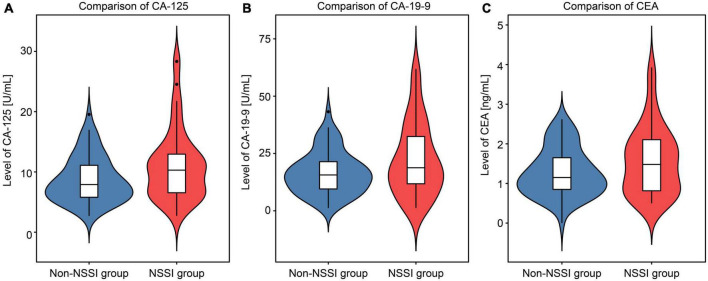
Violin chart comparing the levels of cancer antigen 125 (CA-125), cancer antigen 19-9 (CA19-9), and carcinoembryonic antigen (CEA) between the non-suicidal self-injury (NSSI) and non-NSSI groups.

Compared with those in the non-NSSI group, the average levels of CA-125 (*t* = 2.623, *p* = 0.010), CA19-9 (*t* = 2.504, *p* = 0.014), and CEA (*t* = 2.093, *p* = 0.039) in the NSSI group were significantly higher (Figure 2). No significant differences were found between the two groups in other peripheral blood indices such as routine blood tests, liver and renal function, blood lipid and blood glucose, and thyroid function (*p* > 0.05). See the Supplementary information for the complete data comparison.

### Association between non-suicidal self-injury frequency and tumor markers

**FIGURE 3:**
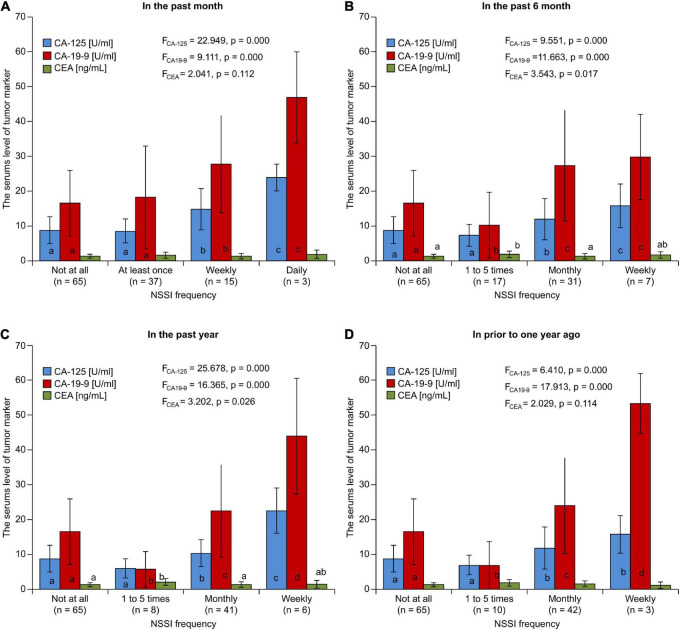
The comparison of serum level of tumor marker grouped according to NSSI frequency in the past **(A)** month **(B)** 6 months **(C)** year **(D)** prior to one year.

Considering the frequency of NSSI behavior, the highest frequency over the past month was 1–5 times (30.83%), followed by monthly in the past 6 months (25.83%), in the past year (34.17%), and prior to 1 year ago (35.00%). In the subgroup analysis stratified by NSSI frequency, there were significant differences in CA-125 and CA19-9 levels between the subgroups (*p* < 0.05) (Figure 3). In further multiple analysis, there were also differences between subgroups. There was no statistically significant difference between the same letters marked group (*p* > 0.05); however, there was a statistically significant difference (*p* < 0.05) between different letters. Spearman correlation analyses showed that NSSI frequency was positively correlated with CA-125 levels (*r*_CA–125_ = 0.302, 0.293, 0.297, and 0.265 for the past month, 6 months, year, and ≥ prior to 1 year ago, respectively; *p* < 0.05) and CA19-9 levels at all time periods assessed (*r*_CA19–9_ = 0.243, 0.283, 0.273, and 0.271 for the past month, 6 months, year, and prior to 1 year ago, respectively; *p* < 0.05); however, no such correlation was found between NSSI frequency and CEA levels (*r*_CEA_ = 0.115, 0.102, 0.092, and 0.092, for the past month, 6 months, year, and prior to 1 year ago, respectively; *p* > 0.05).

### Association of tumor markers with non-suicidal self-injury

**FIGURE 4:**
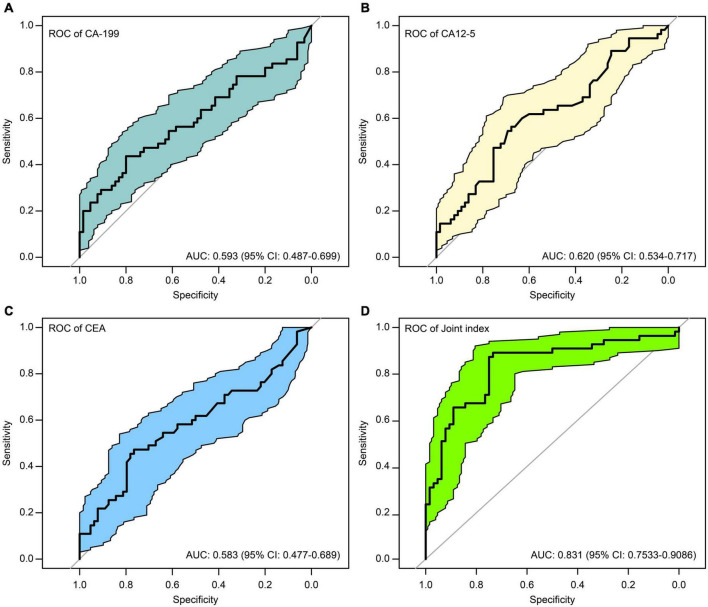
ROC curves of the of cancer antigen 19-9 (CA19-9) **(A)**, cancer antigen 125 (CA-125) **(B)**, carcinoembryonic antigen (CEA) **(C)**, and the joint index **(D)** to predict NSSI.

To explore the correlation between CA-125, CA19-9, or CEA and depressive disorders with or without NSSI, we drew ROC curves for the three tumor markers and verified them using bootstrap analyses. We found that the tumor marker levels had some differential effects on NSSI, with sensitivity and specificity of 0.436 and 0.800 for CA19-9 (Figure 4A), 0.564 and 0.667 for CA-125 (Figure 4B), and 0.473 and 0.766 for CEA (Figure 4C), respectively.

### Construction of a joint index

Due to the unsatisfactory sensitivity and specificity of CA-125, CA19-9, and CEA, we constructed a joint index based on a combination of indicators (Zhu et al., 2015) to increase the sensitivity and specificity. Thus, we used the logistics algorithm to establish a prediction model with CA-125, CA19-9, and CEA, with sex, age, HDRS, and HAMA as candidate predictors. Through these indicators, a joint indicator was constructed.

Joint index = 0.06 × CA-125 + 0.02 × CA19-9 + 1.17 × CEA −0.44 × sex −0.10 × age + 0.17 × total HDRS score −0.005 × total HAMA score.

**FIGURE 5:**
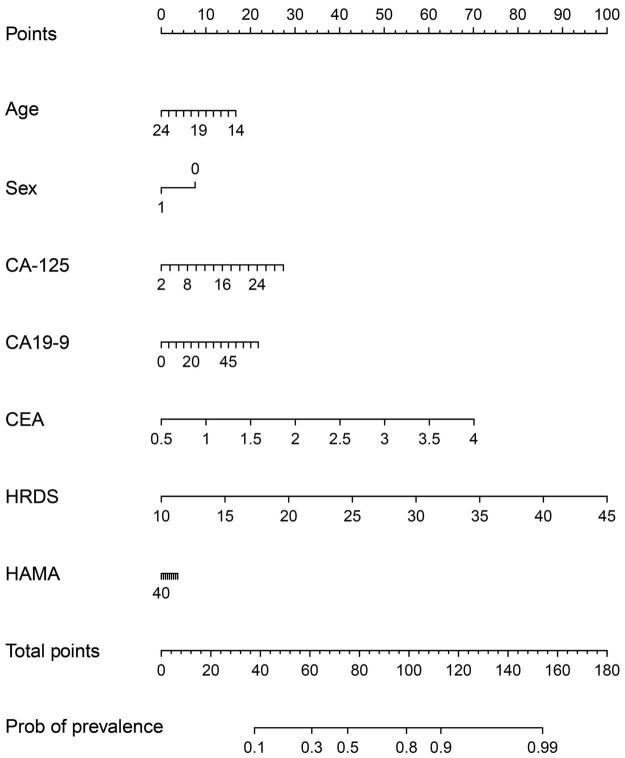
A nomogram predicting the risk of NSSI for adolescents with depressive disorder. The value of each of variable was given a score on the point scale axis. A total score could be easily calculated by adding each single score and, by projecting the total score to the lower point scale were able to estimate the probability of NSSI.

The model is visualized to form a nomogram, as shown in Figure 5, the ROC curve was plotted using this joint index with the occurrence of NSSI as the gold standard (see Figure 4D). The AUC was 0.831 (95%CI:0.7533-0.9086), and the sensitivity and specificity were 0.734 and 0.891, respectively. This indicates a relatively good differential ability of the joint index to predict the occurrence of NSSI among patients with depressive disorders.

## Discussion

This study constructed a joint index consisting of CA-125, CEA, HDRS total score, and age, which had an outstanding ability to recognize NSSI among adolescents with depressive disorders. The AUC was 0.778, and the sensitivity and specificity were 0.703 and 0.769, respectively. To our knowledge, this is the first study to use such an index and provide important evidence for the early identification of NSSI among adolescents with depressive disorders. In the visualized nomogram (Figure 5) generated by the application of the joint index, the scale is marked on the line segment corresponding to each variable, representing the value range of the variable, while the length of the line segment reflects the contribution of this factor to NSSI prediction. Total HRDS scores contributed the most scores in predicting NSSI models (Groschwitz et al., 2015; Zubrick et al., 2016), which was consistent with the results of the present study, suggesting that NSSI is associated with the severity of depressive disorder. Regarding age, we found that with an increase in age, the contribution score of the NSSI prediction model decreased, suggesting that NSSI may gradually decrease with an increase in age. Regarding sex, the corresponding score for females is 10, while that for males is 0, suggesting that females have a higher risk of NSSI (Fox et al., 2015). The total HAMA score was the lowest among the prediction models. Fox et al. (2015) also found that the risk weighting of anxiety factors in NSSI was not high. In addition to HRDS total score, CEA, CA-125, and CA19-9 contributed to the prediction effect. Therefore, it can be considered that CEA, CA-125, and CA19-9 had a better prediction effect on NSSI in adolescents with depressive disorder.

A previous study has reported that NSSI goals mainly include reducing distress, imposing self-punishment, or expressing personal distress (Zhu et al., 2015). According to this theory, the occurrence of NSSI is not intended for suicide purposes; however, NSSI is closely related to SI, AS, and SB (Hawton et al., 2003; Nock et al., 2008; Martin et al., 2010), where NSSI frequency, number of self-injury methods, and hopelessness are the strongest predictors of AS (Victor and Klonsky, 2014; Coppersmith et al., 2017). Notably, NSSI is a risk factor for committing suicide among adolescents with depression (Hawton et al., 2003; Zubrick et al., 2017; Fan et al., 2021; Masi et al., 2021). In particular, female adolescents have a higher risk of committing suicide if they exhibit frequent NSSI behaviors (You and Lin, 2015).

In a study of suicide risk among hospitalized patients with BD and major depression, depression, circularity, irritability, and anxiety personality traits were found to be strongly associated with suicide risk (Baldessarini et al., 2017), which is similar to the findings in this study that NSSI patients have higher symptoms of depression, anxiety and biorhythm disorders. Moreover, the combination of these indicators also has a better predictive effect on NSSI.

Among 120 participants in our study, 55 had NSSI with an incidence rate of 45.83%, which was similar to the proportion reported by Lawrence et al. (2016). Nearly half of adolescents with depressive disorders have NSSI, which is a high-risk factor for suicide. Thus, clinical workers need to pay close attention to NSSI and strengthen suicide prevention education and measures to reduce the occurrence of suicidal behaviors. In addition, NSSI, a symptom newly listed in the DSM-5 (American Psychiatric Association, 2013) that may attract much focus in clinical settings, still needs to be explored. Further studies could provide much needed information regarding the psychological and social functions of NSSI, the pathogenesis of NSSI, and the presence of biomarkers or reliable measurement methods for NSSI.

Palagini et al. (2021) analyzed the predictors of SI and planning among 127 patients with depression or BD and found significant differences in depressive symptoms, feelings of hopelessness, and sleep and social life dysrhythmia between suicidal and non-suicidal groups, feelings of hopelessness, and sleep and social life dysrhythmia played an intermediary role in suicide planning, and sleep and social life dysrhythmia best predicted SI and planning. Some studies have indicated that sleep rhythm disturbances, such as difficulty falling asleep at night, easily awakening after falling asleep, early awakening while feeling fatigued during the day, and hypersomnia, are one of the main clinical manifestations in patients with depressive disorders (Pervaiz et al., 2021). In this study, compared with the non-NSSI group, patients with depressive disorders and NSSI had significantly higher scores for depressive symptoms, insomnia, and feelings of hopelessness. This suggests that NSSI pathogenesis overlaps with SI or SB pathogenesis. Therefore, it is necessary to carefully evaluate biological rhythm disorders, depression degree, and sleep disorders when assessing the risk of suicide and self-injury in patients with depressive disorders. In the present study, the relative effects of sex, age, CA-125, CA19-9, CEA, HRDS, HAMA, and other indicators on NSSI were shown by logistic regression analysis. These indicators are easy to obtain in clinical practice and cause less harm to patients. Clinicians can use the points shown in Figure 5 to evaluate and predict the risk of NSSI in patients with depression, reduce the missed diagnosis of NSSI, and increase the prevention of suicide risk.

Biological rhythm disorders (e.g., dyssomnia) impact the development of psychiatric disorders. For example, sleep deprivation and fatigue can influence learning, memory, and mood, and sleep problems may lead to impaired cognitive function (Deliens et al., 2014; Coogan et al., 2016; Seegers et al., 2016). Sleep-wake cycle disorders are prevalent in patients with depression and BD, who have difficulties falling asleep and easily wake up at night (Rosenblat and Mcintyre, 2017). Genetic studies have reported potential associations between biological rhythms and clock genes (Charrier et al., 2017). These studies show that clock genes participate in biological rhythmicity at the molecular level and alter sleep-wake rhythms during the onset of affective disorders, which, in turn, impairs cognitive function (Sulli et al., 2018; Varinthra and Liu, 2019). In the present study, there were differences between the non-NSSI and NSSI groups in several HDRS and HAMA score factors (Table 2). Given the above research, we speculate that these differences may be a result of a concurrent biological rhythm disorder, which may be related to dysregulated clock genes. Future studies should address the potential relationship between dysregulated clock genes, biological rhythm disorders, and depression with and without NSSI. We further speculated that the differences between the non-NSSI and NSSI groups in sleep disorders, cognition, diurnal changes, hysteresis, and other aspects may be related to biological rhythm disorder, which may be related to the imbalance of biological clock genes. However, further longitudinal studies are required to confirm this hypothesis.

Substantial studies focused on clock genes have revealed that these genes control the daily rhythm of cell proliferation, metabolism, inflammation, and DNA damage responses by disrupting the circadian clock (Coppersmith et al., 2017; Yang et al., 2021). Specifically, night shift work disturbs the circadian rhythm of cancer-related genes by delaying their expression time and reducing repair efficiency during the night when the human body needs DNA repair. This results in more DNA damage and increases the risk of tumor development (Koritala et al., 2021). Such interference underlies the pathogenesis of cancer; thus, individuals with chronic circadian disturbances are predisposed to tumor development (Koritala et al., 2021). Repeated sleep rhythm disorder is a stressor that adversely affects health leading to cancer development and decreased immune function in animal models (Papagiannakopoulos et al., 2016). Another study found that circadian dysregulation affects metabolic function in humans, which might impact antioxidant defenses; thus, biological rhythm disorders alter peripheral blood tumor marker content (Yu and Weaver, 2011). In the present study, we found that compared with the non-NSSI group, adolescents with depressive disorder with NSSI had significantly more sleep rhythm disorders, more severe anxiety and depressive mood, and higher levels of tumor markers (CA-125, CA19-9, and CEA). However, all indicators were within the normal range and all participants were in good physical health; thus, the risk of tumor development was relatively low (Zubrick et al., 2017), and the difference level of tumor markers between NSSI group and non-NSSI group is unlikely to be caused by the tumor itself. According to the above description, combined with a study that found that CA 125 level is related to anxiety and depression (De Moor et al., 2006), we speculate that it may be due to factors such as sleep rhythm disorder mediated by clock genes and anxiety and depression in NSSI group, resulting in the increase of tumor marker level in NSSI (Koritala et al., 2021).

The DSM-5 describes the clinical features and recommended diagnostic criteria of NSSI while pointing out that most people with NSSI are less likely to seek clinical attention and treatment. The proportion of individuals that conceal NSSI behaviors can reach 11–57% (Hasking et al., 2015; Martorana, 2015; Frost et al., 2017); thus, it is difficult to detect NSSI. Other clinical studies have shown that relying on patients’ self-reports is not a true reflection of NSSI frequency (Ammerman et al., 2021). The joint index that was constructed in this study using depression score, tumor markers, and age to identify the occurrence of NSSI provides a simple and valuable method for the timely detection of NSSI.

This study had some limitations. First, the sample size was relatively small, which might have led to selection bias. Second, there was an absence of healthy adolescents in the control group; thus, the study cannot be generalized to the entire adolescent population. Third, adolescence includes the age group of 10–24 years, in which adolescents are in good physical condition with slight physiological effects on tumor markers (Zubrick et al., 2017). However, as patients aged < 14 years were not admitted to our hospital, we could not collect data on adolescents aged 10–13 years. In addition, the hospitals where the researchers were enrolled were all inpatients and were not validated in outpatient and community NSSI adolescent patients. Fourth, the true causality between tumor markers and NSSI remains unclear because of the cross-sectional design of this study without follow-up. Finally, the sensitivity and specificity of our results should be improved by adding more samples and objective physiological indicators. Lack of objective biological indicators of biorhythm disorders, such as major clock genes for measurement and analysis, did not allow us to verify the correlation between biorhythm and tumor markers. Serafini et al. (2014) reported that decreases in basal ganglia and hippocampus volume were more specific for adolescents with unipolar depression, while decreases in corpus callosum volume and increases in deep white matter hyperintensity were more specific for adolescents with BD. Combined with imaging results, it may improve the sensitivity and specificity of the joint index.

In summary, the incidence of NSSI was relatively high in adolescent patients with depressive disorders. In addition, there were significant differences in HAMA scores, HDRS scores, and tumor markers (CA-125, CA19-9, CEA) between the NSSI and non-NSSI groups, and a positive correlation was found between NSSI frequency and CA-125 and CA19-9 levels. By constructing a joint index based on the levels of tumor markers, HDRS scores, and age, we easily and accurately identified NSSI behaviors among adolescents with depressive disorders.

## Data availability statement

The raw data supporting the conclusions of this article will be made available by the authors, without undue reservation.

## Ethics statement

The studies involving human participants were reviewed and approved by the Institutional Review Board of Sir Run Run Shaw Hospital, School of Medicine, Zhejiang University. Written informed consent to participate in this study was provided by the participants’ legal guardian/next of kin.

## Author contributions

WC: conceptualization and writing – review and editing. P-cY and K-mR: methodology. K-mR and Y-hQ: formal analysis. C-mZ, Y-hQ, and LH: investigation. Y-hQ and P-cY: data curation. P-cY: writing – original draft. All authors have read and agreed to the published version of the manuscript.

## Abbreviations

ALC: absolute lymphocyte count
ANC: absolute neutrophil count
ALT: alanine aminotransferase
AFP: alpha fetoprotein
AUC: area under the curve
AST: aspartate aminotransferase
AS: attempted suicides
BD: bipolar disorder
CA-125: cancer antigen 125
CA19-9: cancer antigen 19-9
CEA: carcinoembryonic antigen
CRP: C-reactive protein
EEG: electroencephalography
HAMA: Hamilton Anxiety Rating Scale
HDRS: Hamilton Depression Rating Scale
Hb: hemoglobin
MDD: major depressive disorder
NSSI: non-suicidal self-injury
OSI: Ottawa Self-Injury Inventory
PLT: platelet count
ROC: receiver operating characteristic
RBC: red blood cell count
ALB: serum albumin
SB: suicidal behavior
SI: suicidal ideation
WBC: white blood cell count.
