# Supplementary material for: Tumor markers and depression scores are predictive of non-suicidal self-injury behaviors among adolescents with depressive disorder: A retrospective study
Source: Front Neurosci. 2022 Aug 11;16:953842. doi: 10.3389/fnins.2022.953842 (PMC9403252; doi:10.3389/fnins.2022.953842)
Supplement: Supplementary file 1 [file Table_1.DOCX]

| Item | Non-NSSI group | NSSI group | t | P-value |
| --- | --- | --- | --- | --- |
| Homocysteine (μmol/L) | 13.516±8.362 | 13.916±7.736 | -0.270 | 0.788 |
| Vitamin B12 (pg/mL) | 488.785±229.747 | 531.185±213.765 | -1.034 | 0.303 |
| Folic acid (ng/mL) | 5.300±3.731 | 5.732±3.713 | -0.630 | 0.530 |
| Potassium ion (mmol/L) | 4.050±0.252 | 4.073±0.253 | -0.496 | 0.621 |
| Sodium ion (mmol/L) | 140.923±1.661 | 140.600±1.978 | 0.973 | 0.333 |
| Chloride ion (mmol/L) | 105.554±1.961 | 105.855±2.280 | -0.777 | 0.439 |
| Calcium ion (mmol/L) | 2.364±0.113 | 2.359±0.110 | 0.241 | 0.810 |
| Urea nitrogen (mmol/L) | 4.534±1.379 | 4.380±0.994 | 0.690 | 0.491 |
| Creatinine (μmol/L) | 65.769±15.281 | 65.491±13.008 | 0.106 | 0.915 |
| Glucose (mmol/L) | 4.764±0.427 | 5.037±1.226 | -1.678 | 0.096 |
| Urea nitrogen/creatinine | 0.072±0.021 | 0.069±0.016 | 0.867 | 0.388 |
| Alanine aminotransferase (U/L) | 21.708±20.593 | 20.792±28.246 | 0.203 | 0.839 |
| Aspartate aminotransferase (U/L) | 21.938±12.618 | 20.385±10.652 | 0.709 | 0.480 |
| Alkaline phosphatase (U/L) | 21.938±12.618 | 20.385±10.652 | 0.709 | 0.480 |
| Glutamyl transpeptidase (U/L) | 19.462±12.011 | 20.436±18.412 | -0.348 | 0.728 |
| Total protein (g/L) | 68.489±4.743 | 67.807±4.810 | 0.780 | 0.437 |
| Albumin (g/L) | 42.966±3.641 | 42.033±3.569 | 1.412 | 0.161 |
| Globulin (g/L) | 25.523±3.098 | 25.775±3.289 | -0.431 | 0.667 |
| AlbuminandGlobulinRatio | 1.709±0.262 | 1.659±0.264 | 1.054 | 0.294 |
| Total bilirubin (μmol/L) | 13.282±5.941 | 13.311±6.296 | -0.026 | 0.979 |
| Direct bilirubin (μmol/L) | 2.614±1.284 | 2.525±1.310 | 0.372 | 0.710 |
| Indirect bilirubin (μmol/L) | 10.668±4.796 | 10.785±5.153 | -0.130 | 0.897 |
| Triglyceride (mmol/L) | 1.025±0.555 | 1.098±0.658 | -0.651 | 0.516 |
| Total cholesterol (mmol/L) | 4.357±0.877 | 4.327±0.770 | 0.194 | 0.846 |
| HDL cholesterol (mmol/L) | 1.129±0.235 | 1.087±0.230 | 0.986 | 0.326 |
| Magnesium ion (mmol/L) | 0.888±0.066 | 0.875±0.082 | 0.934 | 0.352 |
| Phosphorus ion (mmol/L) | 1.405±0.203 | 1.408±0.228 | -0.080 | 0.937 |
| Uric acid (μmol/L) | 350.641±91.545 | 353.833±108.525 | -0.173 | 0.863 |
| Lactate dehydrogenase (U/L) | 157.810±60.798 | 154.722±33.755 | 0.332 | 0.741 |
| Osmotic pressure (mOsm/L) | 291.246±3.853 | 290.600±4.241 | 0.874 | 0.384 |
| High sensitivity C-reactive protein (mg/L) | 1.160±3.062 | 1.447±2.106 | -0.588 | 0.558 |
| Total bile acid (μmol/L) | 5.370±4.024 | 4.314±3.225 | 1.566 | 0.120 |
| VLDL cholesterol (mmol/L) | 0.772±0.397 | 0.663±0.387 | 1.504 | 0.135 |
| LDL cholesterol (mmol/L) | 2.346±0.654 | 2.405±0.622 | -0.495 | 0.622 |
| White blood cell count (10^9^/L) | 6.289±1.381 | 6.462±1.483 | -0.659 | 0.511 |
| Red blood cell count (10^12^/L) | 4.693±0.496 | 4.602±0.497 | 1.000 | 0.319 |
| Hemoglobin (g/L) | 139.831±14.837 | 136.836±15.508 | 1.079 | 0.283 |
| Hematocrit (%) | 41.526±4.150 | 40.633±4.253 | 1.162 | 0.248 |
| Mean red blood cell volume (fL) | 88.646±4.368 | 88.420±4.050 | 0.292 | 0.771 |
| Mean hemoglobin (pg) | 29.838±1.672 | 29.765±1.651 | 0.240 | 0.811 |
| Platelet count (10^9^/L) | 221.292±51.166 | 235.055±55.124 | -1.417 | 0.159 |
| Red blood cell distribution width (%) | 12.689±0.868 | 12.664±0.938 | 0.155 | 0.877 |
| Platelet distribution width (%) | 16.168±1.468 | 16.420±2.047 | -0.784 | 0.435 |
| Mean platelet volume (fL) | 8.669±0.958 | 8.660±0.824 | 0.056 | 0.955 |
| Lymphatic percentage (%) | 37.988±9.593 | 36.751±10.088 | 0.687 | 0.493 |
| Percentage of single core (%) | 7.757±1.802 | 7.389±2.163 | 1.016 | 0.312 |
| Percentage of neutrophils (%) | 51.582±9.916 | 53.413±10.910 | -0.963 | 0.338 |
| Percentage of eosinophils (%) | 2.158±1.351 | 2.335±1.558 | -0.663 | 0.509 |
| Percentage of basophil (%) | 0.515±0.259 | 0.527±0.303 | -0.231 | 0.817 |
| Absolute number of lymphocytes (10^9^/L) | 2.318±0.562 | 2.285±0.694 | 0.294 | 0.769 |
| Absolute number of monocytes (10^9^/L) | 0.491±0.165 | 0.467±0.128 | 0.890 | 0.375 |
| Absolute number of neutrophils (10^9^/L) | 3.308±1.282 | 3.526±1.367 | -0.900 | 0.370 |
| Absolute number of eosinophils (10^9^/L) | 0.139±0.089 | 0.147±0.102 | -0.459 | 0.647 |
| Absolute number of basophils (10^9^/L) | 0.023±0.027 | 0.028±0.023 | -1.021 | 0.309 |
| Platelet hematocrit(%) | 0.190±0.036 | 0.202±0.041 | -1.668 | 0.098 |
| Thyroid stimulating hormone (mIU/L) | 1.764±1.111 | 1.733±0.962 | 0.162 | 0.872 |
| Total triiodothyronine (ng/mL) | 1.048±0.577 | 0.966±0.217 | 1.000 | 0.319 |
| Thyroxine (μg/dL) | 5.876±2.270 | 5.906±1.181 | -0.088 | 0.930 |
| Free triiodothyronine (pg/mL) | 3.255±3.505 | 2.827±0.566 | 0.880 | 0.381 |
| Free thyroxine (ng/dL) | 1.010±0.314 | 1.013±0.155 | -0.067 | 0.947 |
| Alpha fetoprotein (ng/mL) | 1.976±1.196 | 2.069±1.239 | -0.419 | 0.676 |
| Total prostate specific antigen (ng/mL) | 0.648±0.457 | 0.829±0.472 | -1.392 | 0.170 |
| Carbohydrate antigen 15-3 (U/mL) | 6.603±3.529 | 6.393±3.631 | 0.236 | 0.814 |

**Supplementary Material**

NSSI = non-suicidal self-injury ; HDL=high-density lipoprotein;VLDL=very-low-density lipoprotein;LDL= low-density lipoprotein

There was no significant difference between the non-NSSI group and NSSI groups in peripheral blood routine, liver function, renal function, blood lipid, blood glucose, serum electrolyte, thyroid function, and other common tumor markers(all p>0.05). It shows that there is no significant difference in the physiological state between the two groups, and they are basically in good health.
